# siMS Score: Simple Method for Quantifying Metabolic Syndrome

**DOI:** 10.1371/journal.pone.0146143

**Published:** 2016-01-08

**Authors:** Ivan Soldatovic, Rade Vukovic, Djordje Culafic, Milan Gajic, Vesna Dimitrijevic-Sreckovic

**Affiliations:** 1 Institute of Medical Statistics and Informatics, Belgrade, Serbia; 2 Mother and Child Health Care Institute of Serbia “Dr Vukan Cupic”, Belgrade, Serbia; 3 Clinic of Gastroenterology and Hepatology, Clinical Center of Serbia, Belgrade, Serbia; 4 Clinic of Endocrinology, Diabetes and Metabolism Disorders, Clinical Center of Serbia, Belgrade, Serbia; 5 School of Medicine, University of Belgrade, Belgrade, Serbia; University of Milan, ITALY

## Abstract

**Objective:**

To evaluate siMS score and siMS risk score, novel continuous metabolic syndrome scores as methods for quantification of metabolic status and risk.

**Materials and Methods:**

Developed siMS score was calculated using formula: siMS score = 2*Waist/Height + Gly/5.6 + Tg/1.7 + TA_systolic_/130—HDL/1.02 or 1.28 (for male or female subjects, respectively). siMS risk score was calculated using formula: siMS risk score = siMS score * age/45 or 50 (for male or female subjects, respectively) * family history of cardio/cerebro-vascular events (event = 1.2, no event = 1). A sample of 528 obese and non-obese participants was used to validate siMS score and siMS risk score. Scores calculated as sum of z-scores (each component of metabolic syndrome regressed with age and gender) and sum of scores derived from principal component analysis (PCA) were used for evaluation of siMS score. Variants were made by replacing glucose with HOMA in calculations. Framingham score was used for evaluation of siMS risk score.

**Results:**

Correlation between siMS score with sum of z-scores and weighted sum of factors of PCA was high (r = 0.866 and r = 0.822, respectively). Correlation between siMS risk score and log transformed Framingham score was medium to high for age groups 18+,30+ and 35+ (0.835, 0.707 and 0.667, respectively).

**Conclusions:**

siMS score and siMS risk score showed high correlation with more complex scores. Demonstrated accuracy together with superior simplicity and the ability to evaluate and follow-up individual patients makes siMS and siMS risk scores very convenient for use in clinical practice and research as well.

## Introduction

Metabolic syndrome (MS) represents a cluster of risk factors, namely elevated glucose levels, high blood pressure, elevated triglycerides, reduced high density lipoproteins and abdominal obesity [1]. During the last century, syndrome changed its name from plurimetabolic syndrome, syndrome x, deadly quartet, insulin resistance syndrome and dysmetabolic syndrome [2, 3, 4]. The first formalized definition of the MS was proposed in 1998 by World Health Organization Working Group on Diabetes [5]. During the following period, different definitions of MS were proposed by World Health Organization, National Cholesterol Education Program Adult Treatment Panel and International Diabetes Federation [6, 7]. A new joint statement in 2009 included participation of the IDF, NHLBI, the World Heart Federation, the International Atherosclerosis Society, and the AHA in an attempt to eliminate the confusion regarding how to identify patients with the syndrome caused by multitude of different definitions used [1]. Clinical diagnosis of the MS defined by the joint statement requires presence of any 3 of the following 5 criteria: elevated waist circumference (defined using population and country specific cut-off values), elevated triglycerides (Tg) (≥1.7 mmol/L), reduced high density lipoprotein (HDL) (<1.03 mmol/L in males and <1.29 mmol/L in females), increased blood pressure (BP) (systolic ≥ 130 mm Hg and/or diastolic ≥ 85 mm Hg) and elevated fasting glucose (≥5.6 mmol/L). Drug treatments for elevated Tg, blood pressure, impaired fasting glucose or reduced HDL are also considered as a positive criteria [1].

During the last decade, parallel to the changes in definitions of the syndrome, an alternative approach to assessment of MS was also developed. Lack of universal definition and the fact that MS was defined as dichotomous variable opened up possibilities for the development of continuous score of the syndrome. Rationale behind the development of the continuous score is straightforward, since loss of information can be expected to occur due to dichotomy of current MS definitions (present/absent). Namely, minimal changes in criteria values could result in classifying patients as having MS or not, although only a negligible change has been made. This could especially be important in patients with borderline values and patients with positive two to three components of the syndrome. So far, most commonly used approaches to calculate a continuous MS score in order to overcome this issue were standardized residuals in linear regression (Z scores) and factor scores of principal component analysis [8, 9, 10, 11, 12]. Although these scoring methods are very precise, both suffer from practical disadvantages. The main issue in the use of these methods is the fact that calculated scores are sample specific and can't be used in any other population (sample). If only one patient is removed from or added to the database, the score must be computed again, due to the fact that it is derived from the regression or principal components of the sample. Also, calculations used in these methods are very complicated and demanding, and require use of specialized statistical/mathematical software. On the other hand, contrary to the dichotomous definition of the MS, which calculates all components equally, continuous metabolic syndrome score derived from principal components calculates loadings from each component separately. Also, minimal changes in values of MS components are measurable and accounted for in continuous scores, while dichotomous approach to the syndrome is unable to account for these subtle changes. So far, no consensus exists in regards to the components of metabolic syndrome score and every researcher calculates score by his own definition or experience. In order to overcome the issues of both dichotomous MS definition and unstandardized continuous MS scores which are noncomparable, there is a need to develop a new, easy to calculate MS score, comparable across different studies and populations.

The aim of the present study was to propose a new, easy to calculate continuous MS score–the siMS score, which could be comparable across different populations and appropriate for everyday use in clinical and research practice.

## Materials and Methods

Study was conducted during the period 2007–2014, in the Clinic of Endocrinology, Diabetes and Metabolism Disorders, Clinical Center of Serbia, Belgrade. The data were retrospectively collected from medical records of 528 subjects, age 7 to 77 years. Ethical committee of Faculty of Medicine, University of Belgrade and Ethical committee of Clinical Center of Serbia, Clinic of Endocrinology, Diabetes and Metabolism Disorders granted approval for present study and waiver for individual written consents on the basis of nonidentifiable use of previously obtained retrospectively collected data. Authors signed written obligation to use these data according to all applicable ethical standards without disclosing the identity of the subjects. Permission for publication of the database containing MS parameters of the subjects without subjects’ personal identifying data was granted by the National Agency for the Protection of Private Data (decision No. 011-00-01340/2015-05).

All subjects underwent interview, physical examination and laboratory analyses. Data regarding interview, namely age, gender, morbidity (diabetes, hypertension, hyperlipidemia, angina pectoris, myocardial infarction, peripheral vascular disease), morbidity in family (same as personal), smoking, alcohol consumption and medication use were collected from medical documentation. Physical examination data included body weight, height, waist circumference, body mass index (BMI) and systolic and diastolic blood pressure. Waist circumference was measured in standing position with non-elastic tape at the middle between the upper point of the bilateral iliac crest and the inferior margin of the rib cage in the horizontal plane at the end of expiration. BMI was calculated by dividing weight in kilograms by square of height in meters. Blood pressure (BP) was measured in sitting position, at the end of interview. Mean arterial pressure (MAP) was calculated using following formula: MAP = Diastolic BP + (Systolic BP—Diastolic BP)/3. Average arterial pressure (AvAP) was calculated using following formula: AvAP = (Systolic BP + Diastolic BP)/2.

Laboratory analyses were performed in the morning after 12 hours of fasting. Cholesterol, HDL and Tg were measured using spectrophotometric method and low-density lipoprotein (LDL) was calculated using Friedewald formula. Two hour oral glucose tolerance test (OGTT) with maximum 75g of glucose was performed according to WHO guidelines [13]. Glucose and insulin levels were assessed at baseline, 60 and 120 minutes after glucose administration. Insulin was measured using chemiluminescent immune assay method (CLIA, Siemens Health Care Diagnostic, USA). Insulin resistance was determined using Homeostatic Model Assessment (HOMA IR) index, calculated as the product of fasting plasma insulin (mIU/L) and fasting plasma glucose levels (mmol/L) divided by 22.5 [14].

Metabolic syndrome was determined using several methods. First, dichotomous MS was defined using latest metabolic syndrome definition [1], as presence of at least 3 of the following 5 criteria: abdominal obesity (waist circumference ≥94 cm in adult male and ≥80 cm in adult female subjects), elevated Tg (≥1.7 mmol/L), reduced HDL cholesterol (<1.03 mmol/L in adult male and <1.29 mmol/L in adult female subjects), increased blood pressure (systolic ≥130 mmHg and/or diastolic ≥85 mmHg) and elevated fasting glucose (≥5.6 mmol/L). Drug treatments for elevated Tg, blood pressure, fasting glucose or reduced HDL were also considered as fulfilled criteria. Dichotomous definition of MS in children and adolescents was also defined using cut off values chosen in accordance with the IDF definition of MS in children and adolescents [15].

Three MS scores were calculated using sum of z scores. First was calculated using standardized z scores for waist circumference, systolic arterial pressure, triglycerides, HDL and glucose regressed for age and gender. In calculation of the second MS score, insulin was used instead of glucose, and in the third MS score, HOMA was used. Triglycerides, insulin and HOMA were transformed using logarithmic transformation in order to obtain normal distribution. Z score for HDL was multiplied by -1. Scores derived from principal component analysis (PCA) were also calculated in several different ways. First PCA derived score was calculated using sum of two factors obtained from the factor analysis of five components, waist circumference, mean blood pressure, triglycerides, HDL and glucose. Instead of glucose, HOMA was used to calculate the second PCA derived score. The score was calculated by summing two factors weighted for the variance explained. Third and fourth PCA derived scores were calculated as first component of previously mentioned factor analyses. Sum of z scores and PCA derived scores were calculated for the whole sample and all scores were calculated for subpopulations of children (<18 years), young adults (18−30 years) and adults (31+ years).

In accordance with the study goal, two scores with identical basis were proposed, one called "siMS score", constructed for the evaluation of continuous metabolic syndrome status, and second called "siMS risk score", developed for risk evaluation of coronary or cerebro-vascular events.

siMS score was calculated using formula (with ref. representing the cutoff values of according variables for the diagnosis of MS):

$$siMS\;score=\frac{2\;x\;Waist}{Height}+\frac{Gly}{ref.}+\frac{Tg}{ref.}+\frac{TAsystolic}{ref.}-\frac{HDL}{ref.(male/female)}$$

In our sample, according to the cutoff values from the joint definition of MS [1], siMS score was calculated using the following formula (with waist circumference and height calculated in cm, glucose, Tg and HDL in mmol/l and systolic blood pressure (TA) in mmHg):

$$siMS\;score=\frac{2\;x\;Waist}{Height}+\frac{Gly}{5.6}+\frac{Tg}{1.7}+\frac{TA\;systolic}{130}-\frac{HDL}{1.02\;or\;1.28\;(male\;or\;female)}$$

siMS risk score was calculated as extension of siMS score using formula:

$$siMS\;risk\;score=siMS\;score\;x\;\left(\frac{Age}{45\;or\;50\;(males\;or\;females)}\right)\;x\;\left(\begin{array}{c} Familly\;history\;of\;cardio\\ or\;cerebro-vasular\;event\\ \left(event=1.2\;,\;else=1\right) \end{array}\right)$$

Reference values of each laboratory, nation or country could be used instead of ones used in the present study. Mean arterial pressure and average arterial pressure were used in first versions of the score, but results were very similar to the results provided by the score calculated using systolic blood pressure, thus systolic blood pressure was used in the final score formula for simplicity purposes. Reference values used in current study are not obligatory for all researchers and can be changed in calculation of siMS and siMS risk scores, in accordance with population or local laboratory reference values.

Age and family history were imputed only in siMS risk score formula. Age of 45 or 50 (for men and women, respectively) is considered as a cutoff for higher incidence of cardio/cerebro-vascular events. In women, the average age of menopause is 50, which is considered as age when risk is beginning to equalize with men in terms of cardiovascular risks [16]. In this formula, coefficient of 1.2 (20% higher risk) was chosen as the average risk of cardio or cerebro-vascular event in people with family history of coronary heart disease or stroke [17].

Results are presented as mean±sd or count (%). Pearson correlation coefficient was used to assess correlation between siMS score and other continuous scores. Variables with non-normal distributions were transformed. Best results were obtained using logarithmic transformation. Data analysis was done in SPSS 20.0 statistical software (IBM corp.). All p values less than 0.05 were considered significant.

## Results

Study group consisted of 528 subjects, 182 male (34.5%) and 346 female (65.5%). Mean age of participants was 36.1±16.2. Mean BMI was 32.5±6.9 kg/m^2^, minimum was 17 kg/m^2^ and maximum 61.8 kg/m^2^. In age group <18 years (n = 73), there were 31 male (42.5%) and 42 (57.5%) female subjects. Mean age in this group was 13.2±2.8 years and mean BMI was 28.1±5.6 kg/m^2^. In age group 18−30 years (n = 161), there were 59 (36.6%) male and 102 (63.4%) female subjects, with mean age of 24.3±3.4 years and mean BMI of 33.4±7.2 (20.5–57.3 kg/m^2^). In age group 31+ (n = 294), there were 92 were male (31.3%) and 202 (68.7%) female subjects, with mean age of 48.2±10.6 years and BMI of 33.1±6.8 kg/m^2^ (20.3–61.8 kg/m^2^). Metabolic syndrome was present in 46.6% of the whole sample, with 24.7% having MS in the youngest group, 42.9% in 18−30 age group and 54.1% in 31+ age group. Out of total 528 patients, data necessary for the calculation of siMS and siMS risk scores were available in 490 and 479 subjects, respectively. Correlation analysis of siMS score with other metabolic syndrome scores, calculated separately for all participants and for age groups is shown in Table 1.

**Table 1:** Correlations between siMS score and other metabolic syndrome scores.

Calculation	Score^†^	siMS score
	No of MS components	.745*
	Sum of Z scores (Gly)	.866*
	Sum of Z scores (HOMA)	.833*
All participants	First component PCA (Gly)	.502*
	Sum of factors PCA (Gly)	.822*
	First component PCA (HOMA)	.758*
	Sum of factors PCA (HOMA)	.850*
	Sum of Z scores (Gly)	.828*
	Sum of Z scores (HOMA)	.807*
Calculated using	First component PCA (Gly)	.784*
age categories	Sum of factors PCA (Gly)^a^	.861*
(<18, 18–30, 31+)	First component PCA (HOMA)	.773*
	Sum of factors PCA (HOMA)^a^	.803*

*p<0.001

^†^in bracket is parameter used for calculation: Glucose (Gly) or insulin resistance (HOMA)

^a^Only one factor extracted in 18–30 age group

Correlation analysis revealed high correlation of siMS score with all continuous metabolic syndrome scores calculated for all age groups together (0.758−0.866) except 0.502 for first component PCA, and for all scores calculated separately for age groups (0.773−0.861).

ROC analysis was carried out to in order to evaluate discrimination power of continuous scores. For the simplicity purposes, only three scores were analyzed. First score was derived from sum of z scores of conventional metabolic syndrome components adjusted for age and gender and calculated on whole sample. Second score was derived from principal component analysis (same metabolic syndrome components as in previous score) with sum of factor scores weighted for explained variability in total sample. Third score was siMS score. Dichotomous metabolic syndrome variable was used as a state variable. Area under the curve was 0.914 (95% CI 0.889–0.938; p<0.001) for sum of z scores for the whole sample, 0.895 (95% CI 0.868–0.922; p<0.001) for sum of scores derived from PCA and 0.926 (95% CI 0.903–0.950; p<0.001) for siMS score. Although all scores were significant, and with similar coefficients, siMS score had the highest area under the curve.

Table 2 illustrates a sample of five patients with calculated siMS and siMS risk scores. Table 2 presents how siMS score can change differently in regards to siMS risk score, because the risk score accounts for time component as well as heredity.

**Table 2:** Five patients with calculated siMS scores and siMS risk scores within time.

Patient^†^	Time	Waist	Height	TA_systolic_	HDL	Tg	Gly	Age	Family history	siMS score	siMS risk score
1 (f)	Baseline	132	167	120	0.91	2.49	5.7	20	+	**4.28**	**2.05**
	After 2 years	120	168	120	1.45	1.31	5.2	22		**2.92**	**1.54**
2 (m)	Baseline	104	190	140	1.03	2.09	5.5	30	+	**3.37**	**2.69**
	After 7 years	103	190	140	1.1	1.9	5.4	37		**3.17**	**3.12**
3 (f)	Baseline	99	161	130	1.17	2.88	5	53	-	**3.90**	**4.14**
	After 1 year	93	161	120	1.30	1.90	5.3	54		**3.13**	**3.38**
4 (f)	Baseline	95	161	160	1.19	1.8	5.2	42	+	**3.46**	**3.49**
	After 10 year	90	161	130	1.20	1.60	5.0	52		**3.01**	**3.76**
5 (m)	Baseline	141	180	125	1.25	1.58	5.3	36	-	**3.18**	**2.54**
	After 3 years	131	180	110	1.2	1.4	5.4	39		**2.91**	**2.52**

^†^ Gender: m-male f-female

As shown in Table 2, siMS score depends solely on MS components while siMS risk score is time dependent. SiMS score can be reduced over time in particular subject, but in the same patient, siMS risk score can be increased due to the change in time component of the score.

Our score (siMS risk) was compared with a sex-specific multivariable 10-year risk factor algorithm, Framingham score. Correlation table with different samples (total sample, 18+, 30+ and 35+) revealed significant correlation between siMS risk score and Framingham score (Table 3). Also, logarithmic transformation of the Framingham score was done to eliminate extreme values and to obtain normal distribution.

**Table 3:** Correlation between siMS risk score and Framingham score.

SAMPLE	Log Framingham
Age 18+	.835*
Age 30+	.707*
Age 35+	.667*

*p<0.001

All correlations were repeated with siMS score and risk score calculated with Tg divided by 2 times referent value (2*1.7) and obtained results revealed approximately 0.05 higher correlation coefficient. Having in mind the importance of simplicity in clinical and research practice, we retained the first definition of siMS score.

## Discussion

It has been emphasized many times that current dichotomized definition of MS results in loss of information, especially regarding minimal changes in borderline biological or measured test values [18]. In order to overcome this, several continuous MS scores were developed [19, 20]. However, these continuous scores suffer from numerous limitations. All these scores are sample-specific, and individual score of a single patient cannot be same in two different studies. The only way to compare mean scores derived in one study to scores derived from another study is to have similar distribution of data and similar measures of central tendency and variability, which is made even more difficult considering the fact that different researchers used different variables and statistical procedures in calculation of MS scores (principal components, standardized z scores with different regressors etc.). Also, weighting of each individual variable to the score is considered equal in z score approach while in factor analysis and principal component analysis, the loadings of each variable are calculated independently [21]. Therefore, there is a need for developing universal criteria for calculation of score which could be used and compared in clinical and research practice.

In previous studies aimed at developing continuous MS score, most researchers used similar variables to ones used in the present study to calculate the score. Recent studies demonstrated single underlying factor structure of metabolic syndrome and stability over time period of few years [22, 23, 24, 25]. Factor core can be calculated in several ways, either as a sum of factor scores weighted by explained variability or as a single factor [20, 26]. There are no standardized variables defined for calculating factor scores using PCA. Most researchers used four factors with HOMA or insulin instead of glucose, triglycerides to HDL ratio are also calculated to reduce number of factors, and blood pressure is calculated either using systolic blood pressure or MAP. Confirmatory analyses in these studies revealed higher factor loadings of waist circumference and glucoregulation measures than loadings of blood pressure and lipids [22, 26]. Lower factor score loading of glucose was established in one study, but only one study in subjects of Asian ethnicity demonstrated this finding [27].

Results of our study indicate that the developed siMS score correlates highly with other continuous scores, but is much easier to calculate, can be applied to individual patients (compared to the other continuous MS scores which require groups of patients), and can also be used for follow-up of a single patient. Correlation analysis of siMS score and other scores revealed high agreement between these scores, with correlation coefficients higher than 0.8. Since age range in our study group was very wide (70 years), calculations of continuous scores were also performed in separate age categories, to reduce possible age bias. Correlation coefficients were similar to whole samples, which further confirms the finding of siMS score being accurate despite the age of participants. ROC analysis revealed that all scores had high discrimination power for presence/absence of metabolic syndrome. This was expected since all scores are calculated from MS components. High correlation between siMS score and other scores resulted in similar area under the curve.

In an addition to siMS score, siMS risk score was derived from siMS score in order to quantify the risk of cardiovascular events, using the same basis of easy to calculate formula. siMS risk score is siMS score with age and heredity component accounted for. Having in mind that present study is cross-sectional, no outcome correlation with risk score could be performed as in studies with follow up [28, 29, 30]. However, correlation of siMS risk score with the best available and already outcome validated risk scores was possible, for which purpose the risk score calculated by D'Agostino et al. was chosen [28]. Results from reference score were log transformed to obtain normal distribution and linear relationship. As shown in results, our score shows very high correlation with the referent score and is much simpler and easier to calculate. Absence of follow-up in present study precludes us from calculating the cut-off value, which should be addressed in further longitudinal studies. Also, siMS risk score should prove useful in follow-up of individual patients, having the possibility of calculating the change in risk over time. As stated, the main limitation of present study is that it’s cross-sectional in character, thus the validity of siMS risk score was based on correlation of our score with other scores. However, our study is strengthened by demonstrated high correlation of results obtained using siMS and siMS risk scores with the results obtained using the currently best available continuous risk scores in a large sample, which provides a base for further research and validation in longitudinal outcome studies.

In conclusion, search for balance between simplicity and accuracy resulted in the development of siMS score and siMS risk score. The presented siMS and siMS risk scores have high correlation with best available (and highly complex) scores, while being much easier to calculate without the need for advanced software. Therefore, siMS and siMS risk scores are appropriate for use in everyday clinical practice and research as well for evaluation and follow-up of individual patients.

## Supporting Information

S1 Application Android application for score calculation. (7Z)

S1 Calculator Score calculator in Excel spreadsheet. (XLSX)

S1 Database (XLSX)
